# Outcome after polytrauma in a certified trauma network: comparing standard vs. maximum care facilities concept of the study and study protocol (POLYQUALY)

**DOI:** 10.1186/s12913-016-1468-5

**Published:** 2016-07-11

**Authors:** Michael Koller, Antonio Ernstberger, Florian Zeman, Julika Loss, Michael Nerlich, on behalf of the Trauma Network Eastern Bavaria (TNO), Bauer, Lehner, Cedl, Walter, Schandelmaier, Maurer, Wick, Barz, Obermeier, Ganslmeier, Bay, Mehlhorn, Schmickal, Obernhuber, Füchtmeier, Nerlich, Schneider, Oedekoven, Schäfer, Albersdörfer, Rieger, Schmid, Neubaruer-Grartzke, Walter

**Affiliations:** Center for Clinical Studies, University Medical Center Regensburg, D-93042 Regensburg, Germany; Department of Trauma Surgery, University Medical Center Regensburg, Regensburg, Germany; Institute of Epidemiology, Medical Sociology, University of Regensburg, Regensburg, Germany

**Keywords:** Health services research, Cohort study, Management of polytrauma, Multiple trauma, Comprehensive trauma network, Outcome research, Patient- reported outcomes, Practice guidelines, Qualitative analysis, Analysis of barriers and facilitators, Trauma register

## Abstract

**Background:**

The aim of this study is to evaluate the performance of the first certified regional trauma network in Germany, the Trauma Network Eastern Bavaria (TNO) addressing the following specific research questions: Do standard and maximum care facilities produce comparable (risk-adjusted) levels of patient outcome? Does TNO outperform reference data provided by the German Trauma Register 2008? Does TNO comply with selected benchmarks derived from the S3 practice guideline? Which barriers and facilitators can be identified in the health care delivery processes for polytrauma patients?

**Method/design:**

The design is based on a prospective multicenter cohort study comparing two cohorts of polytrauma patients: those treated in maximum care facilities and those treated in standard care facilities. Patient recruitment will take place in the 25 TNO clinics. It is estimated that *n* = 1.100 patients will be assessed for eligibility within a two-year period and *n* = 800 will be included into the study and analysed.

Main outcome measures include the TraumaRegisterQM form, which has been implemented in the clinical routine since 2009 and is filled in via a web-based data management system in participating hospitals on a mandatory basis. Furthermore, patient-reported outcome is assessed using the EQ-5D at 6, 12 and 24 months after trauma.

Comparisons will be drawn between the two cohorts. Further standards of comparisons are secondary data derived from German Trauma Registry as well as benchmarks from German S3 guideline on polytrauma.

The qualitative part of the study will be based on semi-standardized interviews and focus group discussions with health care providers within TNO. The goal of the qualitative analysis is to elucidate which facilitating and inhibiting forces influence cooperation and performance within the network.

**Discussion:**

This is the first study to evaluate a certified trauma network within the German health care system using a unique combination of a quantitative (prospective cohort study) and a qualitative (in-depth facilitator/barrier analysis) approach. The information generated by this project will be used in two ways. Firstly, within the region the results of the study will help to optimize the pre-hospital and clinical management of polytrauma patients. Secondly, on a nationwide scale, influential decision-making bodies, such as the Ministries of Health, the Hospital Associations, sickness funds, insurance companies and professional societies, will be addressed. The results will not only be applicable to the region of Eastern Bavaria, but also in most other parts of Germany with a comparable infrastructure.

**Trial registration:**

VfD_Polyqualy_12_001978, 10.Jan.2013; German Clinical Trials Register DRKS00010039, 18.02.2016.

## Background

To date, chronic diseases have primarily been investigated in the context of health services research. One of the main reasons for this may be the wide availability of accessible data in the elective case situation. Acute care situations are much more challenging in terms of study management and data acquisition. Therefore this important segment of health care is definitely under-researched. This fact represents a striking contrast to the enormous amount of health care costs that are spent in acute and emergency care settings. Of particular importance is major multiple trauma (used synonymously with the term “polytrauma”) which causes higher socioeconomic burden than oncological and cardiovascular diseases [1, 2]. Consequently, the World Health Organization (WHO) has identified trauma as a key issue in future health care [3].

### Current state of research

The German Trauma Society (Deutsche Gesellschaft für Unfallchirurgie, DGU) started a nationwide multicentre study based on voluntary participation of trauma centres in 1993. This registry data collection currently contains some 160.000 cases with over 130 single items per patient until December 2013. The aim of this trauma registry was to analyse the performance of individual trauma centres, including the pre-hospital management, the clinical care setting, and outcome data. The main results of the Trauma Register DGU® [4] were published and served as the empirical and conceptual basis for the “White Book of the Severely Injured Patient” (2nd ed, 2012, [5]).

The White Book delineated the creation of a nationwide system of regional trauma networks. Similar to the American trauma system, a ranking of trauma facilities was introduced where all participating hospitals were classified into basic, (“Lokales Traumazentrum”, comparable to US level III), standard (“Regionales Traumazentrum”, comparable to US level II), and maximum (“Überregionales Traumazentrum”, comparable to US level I) care facilities. Strict rules for patient transfer were introduced with the aim to "get the right patient at the right time to the right hospital". According to the White Book the maximum transportation time for a major trauma patient from the scene should not exceed 30 minutes. The patient has to be transported to the next available standard or maximum care facility. If this time interval cannot be adhered to (either by ground or air transportation), the nearest basic trauma facility has to initially stabilize the patient and then transfer him/her as soon as possible to the next higher level of care institution. All hospitals within a region were audited and the network was not certified until an agreement on cooperation and uniform patient documentation was signed and the German S3 guideline on polytrauma care [6] has been implemented.

The effects of this major innovation in the German health care system cannot be predicted. Quite generally, it remains unclear whether trauma networks indeed improve the quality of care. More recently, trauma networks or trauma systems have been established in a number of different countries. Thus far, the reports on the results on an international level have been controversial. The Major Trauma Outcome Study in the USA, a trauma registry with more than 150.000 patients [7] has demonstrated the superiority of treatment of multiple injured patients in dedicated trauma centres [8]. In a Dutch study [9], the introduction of a regional trauma system reduced mortality and changed admission rates for all types of trauma. Other reports from the United Kingdom and from Switzerland could not show any improvement for mortality [10, 11]. The authors reached different conclusions in their respective health care settings due to the difference in the health care systems.

Based on these various philosophies the design of trauma centres differs greatly between countries with respect to size and catchment area (e.g. North America: centralization into a few maximum care facilities, Germany: decentralized health care system with many small to medium-sized facilities). Therefore it is difficult to apply the results of foreign studies to the German situation. Improving the quality of care also includes a profound understanding of care processes [12], which is especially true for the treatment of polytrauma patients in the acute period, due to the critical timeframe and the multidisciplinary co-operation required [13]. The “blueprint” is a quality management tool derived from the service industry, which structures and visualizes complex, customer-centred service processes and helps identify critical points (“moments of truth”) along the service process and within the interaction of different stakeholders [14, 15]. The blueprint method has been successfully transferred to patient-centred health care processes by some authors, e.g. with regard to mammography performance or outpatient care of hypertension [16, 17]. The method has not yet been applied to the acute care situation.

### Aim of the study

The aim of the present project is to evaluate the performance of the first certified regional trauma network in Germany, the Trauma Network Eastern Bavaria (TNO). In order to assess *qual*it*y* of care for *poly*trauma (*POLYQUALY*) the following specific research questions will be addressed:

- Do standard and maximum care facilities produce comparable (risk-adjusted) levels of patient outcome?
- Does network formation like the TNO outperform reference data provided by the German Trauma Register DGU^®^ 2008?
- Does TNO comply with selected benchmarks derived from the S3 practice guideline?
- What barriers and facilitators in the process of delivering health care to major trauma patients can be identified? How can these factors explain the results in patient outcome?

We hypothesize that the quality of care and therefore the outcome will be equal in hospitals with standard care in comparison to hospitals with maximum care facilities in a region if all hospitals are organized into a network structure that allows mutual support including the use of latest technologies such as telemedicine consultation. Furthermore, we hypothesize that TNO outperforms German Trauma Register DGU^®^ 2008 reference data and that the quality of care in the standard and maximum care facilities of TNO complies with guideline-derived benchmarks. In case our hypotheses hold true the qualitative barriers and facilitators analysis will be able to explain what the driving forces were that made this favorable results possible. In case the study results contradict our hypotheses the barriers and facilitators analysis can explain the hindering forces and may show ways how to improve the health care situation in the region.

The present project will also consider gender aspects. The majority of trauma patients (about 70 %) are male. In this study males and females will be enrolled according to the actual incidence of trauma events. After major trauma, quality of life differs between genders. Women have a 2.5 fold greater risk than men for developing psychological problems following major trauma and intensive care treatment (post-traumatic stress disorder, PTSD) [18, 19]. In order to address these gender-specific differences, quality of life outcomes will be compared between males and females.

## Methods/design

### Study design

**Fig. 1:**
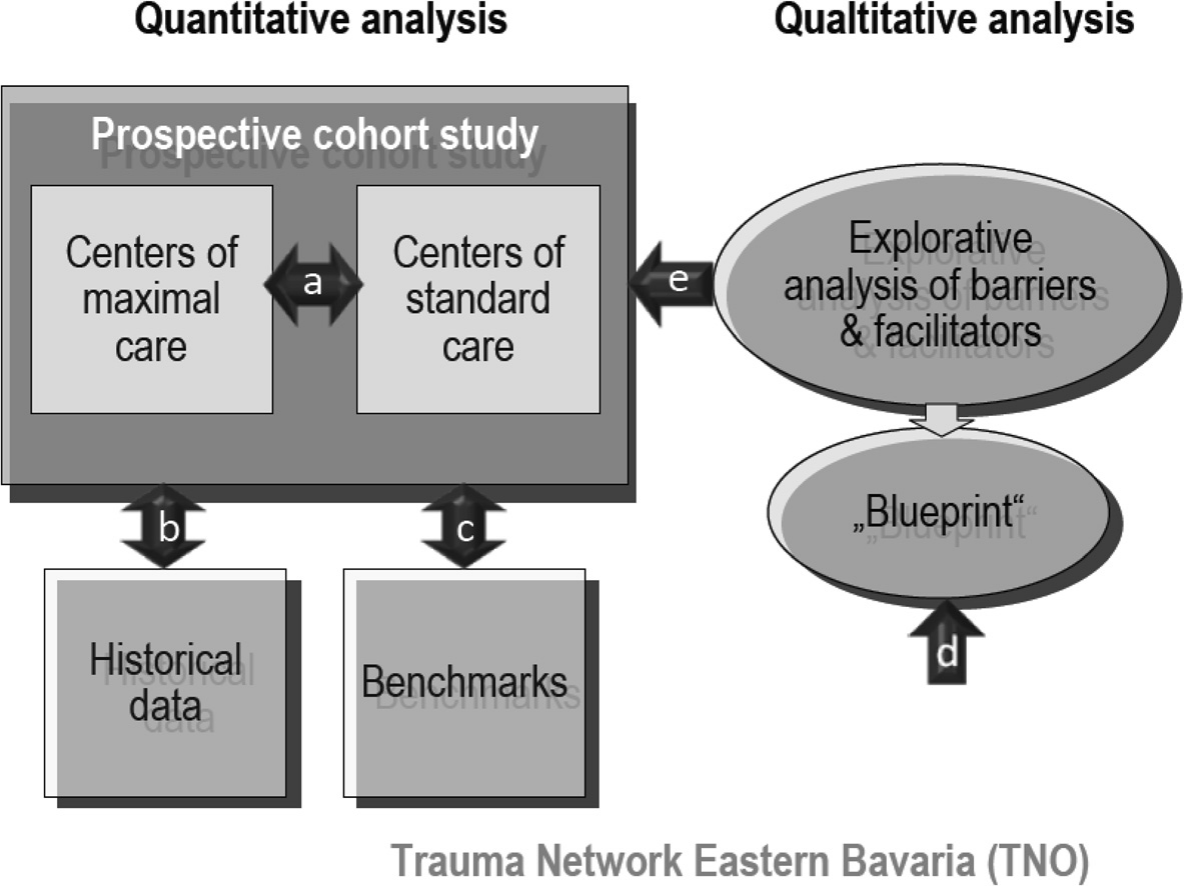
Study design

The study design includes a prospective quantitative analysis and a related explorative qualitative analysis (see Fig. 1).

The *prospective multicentre study* will (a) compare two cohorts of major trauma patients, those treated in maximum care facilities vs. those treated in standard care facilities, (b) compare results of the two cohorts with historical controls derived from the German Trauma Register DGU^®^ 2008 reference data and (c) compare results of the two cohorts with benchmarks including 5 outcome standards derived from the S3 guideline: risk adjusted morality (<15 %), door to CT-time (<20 min), ventilator free days (>22 days), ICU free days (>20 days), GOS (>80 % excellent and good).

The *qualitative part of the study* will (d) develop a service mapping tool (“blueprinting”) derived from quality management in the service industry in order to structure and visualize the care process for major trauma patients in the TNO, and (e) employ focus group discussions for identifying barriers and facilitators during the process, which can serve to explain why benchmarks are or are not met. This design allows for a comprehensive evaluation of TNO. In addition, the triangulation of methods provides for a profound understanding of the processes involved in acute major trauma care as well as the causal relationship between processes, structures and patient outcome. Whereas the quantitative study supplies data of patient outcome, and allows for comparisons between different health care standards plus the comparison of these results to benchmarks, the additional qualitative data provide an aid to interpretation and thus help in using the data for improvement of care processes and quality of care.

### Study population and sampling

Patients will be recruited within the 25 hospitals that comprise TNO (Table 1). Inclusion criteria for the *prospective cohort study* are: major trauma ISS ≥ 16 (Injury Severity Score), treatment in any of the care facilities within the trauma network TNO, no age limitations. Exclusion criteria are ISS < 16, care outside TNO. Based on current register data it is expected that *n* = 1.100 patients will be assessed for eligibility and n = 800 be included and analyzed. For the *qualitative analysis* of barriers and facilitators, focus groups will be conducted with representatives of different professional groups involved in the TNO including emergency physicians, paramedics, dispatch centre staff, emergency room staff, trauma surgeons, rehabilitation staff and social workers. The focus group methodology can be transferred to other trauma networks and can be spread throughout Germany.

**Table 1:** Trauma Network Eastern Bavaria / recruiting centres

Centre	Level of care
University Hospital Regensburg	Maximum Care Facility
Krankenhaus der Barmherzigen Brüder Regensburg	Maximum Care Facility
Klinikum Amberg	Standard Care Facility
Klinikum Eggenfelden	Standard Care Facility
Klinikum Deggendorf	Standard Care Facility
Klinikum Landshut-Achdorf	Standard Care Facility
Klinikum Neumarkt	Standard Care Facility
Klinikum Passau	Standard Care Facility
Klinikum Straubing	Standard Care Facility
Klinikum Weiden	Standard Care Facility
Krankenhaus Bogen	Basic Care Facility
Krankenhaus Burglengenfeld	Basic Care Facility
Krankenhaus Cham	Basic Care Facility
Krankenhaus Dingolfing	Basic Care Facility
Krankenhaus Freyung	Basic Care Facility
Krankenhaus Kelheim	Basic Care Facility
Krankenhaus Mallersdorf	Basic Care Facility
Krankenhaus Marktredwitz	Basic Care Facility
Krankenhaus St. Josef Regensburg	Basic Care Facility
Krankenhaus Schwandorf	Basic Care Facility
Krankenhaus Viechtach	Basic Care Facility
Krankenhaus Vilsbiburg	Basic Care Facility
Krankenhaus Vilshofen	Basic Care Facility
Krankenhaus Waldkirchen	Basic Care Facility
Krankenhaus Zwiesel	Basic Care Facility

### Data collection

Clinical information will be collected using the TraumaRegister^QM^ form. This quality management (QM) form underwent a rigorous development process and has been issued by the German Trauma Society. TraumaRegister^QM^ is a one-page, 40-item short form that includes among other things the following contents: date and site of referral, type of trauma, pre-hospital management, Glasgow Coma Scale (GCS), in-hospital management, ICU management and outcome at discharge (Glasgow Outcome Scale, GOS). A number of validated scores can be delineated from this documentation, such as AIS, ISS, TRISS, RISC, [20] for evaluation of adjusted outcome. TraumaRegister^QM^ will be completed by the participating hospitals via a web-based system. Data will be transferred to AUC (Akademie der Unfallchirurgie) and from there to the Centre for Clinical Studies Regensburg.

**Fig. 2:**
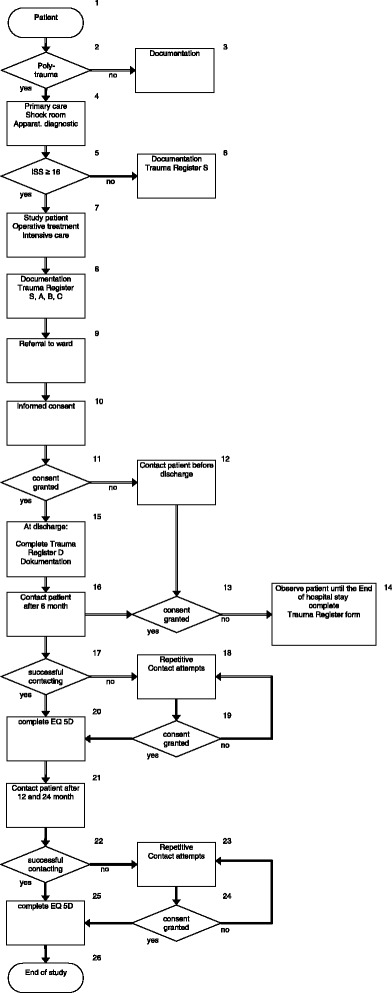
Algorithm patient flow

Another data source which will be used as a historical control is the German Trauma Register DGU^®^ reference data base. Quality of life will be assessed using the EQ-5D form which is a short form widely used in large scale cohort studies. This instrument underwent rigorous psychometric testing, is part of the trauma-specific QL measurement system POLO-Chart [21] and generates indices that can be used for further socio-economic analyses. A German version and population-based German reference data are available. EQ-5D assessment will be performed 6, 12 and 24 months after trauma (Fig. 2). Assignment to the respective clinical information documented in TraumaRegister^QM^ will be accomplished via an index number.

In the course of the qualitative part of this project the semi-standardized interviews and focus groups will be conducted face-to-face, audio-taped and transcribed verbatim. For the analysis of barriers and facilitators, the developed blueprint will be used as a basis for focus group discussions.

### Methods against bias

The use of TraumaRegister^QM^ is mandatory for participating hospitals via the TNO contract and hospital staff will be trained to complete the form via a web-based system. Non-compliant hospitals are easily detectable. Assistance with data collection and checks on plausibility will be provided by a data manager hired for this study and affiliated with the Centre for Clinical Studies at the University Hospital Regensburg. Thus, a near complete (>95 %) documentation of all major trauma patients with a minimum of missing values for the two-year recruitment period can be expected. The quality management of the *qualitative data* collection and analysis will be performed according to the standards set by [22]. Among others, it is planned to utilize “respondent validation” by presenting the results and discussing them with interviewees and focus group members in interactive workshops.

### Biostatistical concept / statistical analysis

*Prospective cohort study*: Statistical analyses of the prospective data will primarily dwell on descriptive statistics using counts, percentages, means/standard deviations, medians/interquartiles/ranges and confidence intervals. In order to interpret descriptive results they will be compared with secondary data from the Trauma Register DGU^®^ reference data bank 2008 and the benchmarks derived from the S3 guideline on major trauma. A logistic regression will be performed with 30 day mortality as the dependent variable and the ISS and TRISS scores as covariates. A p value of < 0.05 will be considered statistically significant. All other statistical tests will be chosen according to the scale level of the variable and the number of variables used in the model (t-test, Mann–Whitney U-Test, chi-squared test, risk-adjusted multivariable regression models) and will be performed in an explanatory manner, leaving alpha at < 0.05.

*Qualitative study*: Interview and focus group transcripts will be critically examined using thematic content analysis [23]. Themes will be identified using a grounded conceptualisation process [24]. Transcripts will be repeatedly read before and after coding to ensure proper categorisation of data. For the blueprint, the information on the health care process will be visualized as a two-dimensional picture: The horizontal axis represents the chronology of actions by the patient and the health care professional. The vertical axis distinguishes between different areas of actions, i.e. direct interactions between patient and professional, professional actions visible and invisible to the patient, support processes, and planning, managing and controlling activities [15, 17].

### Quality assurance and safety

Only authorized persons in the participating hospitals will have access to the data base, identifying themselves with user codes and passwords. Data transfer from peripheral data capture institutions to the data analysis centre complies with security and safety standards analogue to SSL coding. Data of the prospective study will be anonymised. Nevertheless, the combination of a set of variables documented in each TraumaRegister^QM^ form (data of referral and reception, referring and receiving hospital, age, gender, diagnosis) will allow for matching of two or more forms in case a patient is being referred from one hospital to another. TraumaRegister^QM^ data will be matched with respective EQ-5D forms using a unique index number. The completeness and plausibility of forms and documentation will be cross-checked by the data manager.

### Ethical considerations, funding, and trial registration

Due to the observational nature of the *prospective study* no additional invasive diagnostic or therapeutic regimens are required. Data will be collected anonymously. The documentation and use of data generated with the TraumaRegister^QM^ form has been contracted with the German Trauma Society. Patients will be asked to give informed consent to take part in EQ-5D outcome evaluation.

*Qualitative part of the study*: In the transcripts, the names of the participants (interviewees and focus group members) will be de-identified, and pseudonyms will be used for analysis and data presentation. All participants will be asked consent about audiotaping and transcribing their statements and are free to opt out at any given time.

The POLYQUALY study has been approved by the Ethics Committee of the University of Regensburg (reference number 10-101-0077), is funded by a grant from the German Federal Ministry of Education and Research (reference number 01GY1153), and is registered in the data base of the German Network of Health Services Research (www.versorgungsforschung-deutschland.de/) reference number VfD_Polyqualy_12_001978, date 10.Jan.2013, and in the German Clinical Trials Register DRKS00010039, 18.Feb.2016.

## Discussion

The health condition under investigation involves a large number of patients and causes tremendous socioeconomic costs and long-lasting health problems [25–28]. The study will take place in a circumscribed area of 20.000 km^2^ (Eastern Bavaria is composed of the districts Oberpfalz and Niederbayern), including 2.2 million inhabitants and is representative of other regions in Germany. The 25 participating hospitals are organized in a certified trauma network according to the standards of the German Trauma Society. The reason why the region of Eastern Bavaria was successful in establishing the first trauma network in the German health care system were the existence of a large number of standard and basic care facilities and the construction of a maximum care university hospital in the 1990s which is the newest and one of the most advanced institutions of its kind in Germany. It was the philosophy of the founders of the university hospital from the very beginning to serve as a nucleus of high-performance medicine in the region and to establish strong ties with the surrounding hospitals.

This is the first study to evaluate a certified trauma network within the German health care system using a unique combination of a quantitative (prospective cohort study) and a qualitative (in-depth facilitator/barrier analysis) approach. The information generated by this project will be used in two ways.

Firstly, within the region the results of the study will help to optimize the pre-hospital and clinical management of major trauma patients. The qualitative part of the study will yield results on barriers and facilitators of health care and may serve as a hands-on tool for quality improvement, which can easily be understood and used by the different stakeholders. The TNO quality circle will be used as the forum to convey and discuss this information and to initiate appropriate action.

Secondly, on a nationwide scale, influential decision-making bodies, such as the Ministries of Health, the Hospital Associations, sickness funds, insurance companies and professional societies, will be addressed. The results will not only be applicable to the region of Eastern Bavaria, but also in most other parts of Germany with a comparable infrastructure. Results will be reported and discussed in depth with the German Trauma Society (DGU) in the context of the White Book and the S3-guideline on the management of polytrauma patients.

Therefore, we expect that this study will contribute substantially to the body of knowledge on polytrauma patients and have an impact on the health care system as well as on the individual patient.

## Abbreviations

AIS, abbreviated injury scale; AUC, Akademie der Unfallchirurgie (College of Trauma Surgery); CT, computed tomography; DGU, Deutsche Gesellschaft für Unfallchirurgie (German Trauma Society); GCS, glasgow coma scale; GOS, glasgow outcome scale; ICU, intensive care unit; ISS, injury severity score; POLO-Chart, polytrauma outcome (POLO) chart; POLYQUALY, quality of care for polytrauma; RISC, revised injury severity classification; TNO, traumanetzwerk Ostbayern (Trauma Network Eastern Bavaria); TRISS, trauma injury severity score; WHO, World Health Organization.
